# A New Definition of Peridynamic Damage for Thermo-Mechanical Fracture in Brittle Materials

**DOI:** 10.3390/ma19020234

**Published:** 2026-01-07

**Authors:** Sitong Tao, Fei Han

**Affiliations:** Department of Engineering Mechanics, Dalian University of Technology, Dalian 116024, China

**Keywords:** peridynamics, thermo-mechanical model, peridynamic damage

## Abstract

A thermo-mechanical fracture modeling is proposed to address thermal failure issues, where the temperature field is calculated by a heat conduction model based on classical continuum mechanics (CCM), while the deformation field with discontinuities is calculated using the peridynamic (PD) model. The model is calculated using a CCM/PD alternating solution based on finite element discretization, which ensures the calculation accuracy and facilitates engineering applications. The original PD model defines damage solely based on the number of broken bonds in the vicinity of the material point, neglecting the distribution of these bonds. To address this limitation, a new definition of the PD damage accounting for both the number of broken bonds and their specific distribution is proposed. As a result, damage in various directions can be captured, enabling more realistic thermal fracture simulations based on a unified mesh discretization. The effectiveness of the proposed model is validated by comparing numerical examples with analytical solutions. Moreover, simulation results, including a thermal shock case with a transient temperature field, demonstrate the model’s ability to aid in understanding the initiation and propagation mechanisms of complex thermal fractures.

## 1. Introduction

With the rapid development of industry, more and more high-temperature concrete and metal materials are used. However, unpredictable thermal deformation and stress, often resulting from uneven temperature distributions or inconsistent thermal expansion coefficients, can ultimately lead to structural failure. In order to ensure the safety and reliability of the structures, it is necessary to analyze the thermal deformation and thermal stress that may occur in the structures. However, experiments are complex, and it is costly to reproduce the high temperature and high pressure environment. Therefore, numerical simulation is an alternative approach to help understand the mechanisms of the thermo-mechanical coupling response of a structure. Therefore, an efficient and accurate numerical simulation method is necessary.

CCM is widely used for continuous thermo-mechanical problems, but its partial differential governing equation cannot handle discontinuous issues such as thermal fracture. To address this, several numerical methods have been developed, including the extended finite element method (XFEM) [1], phase-field fracture method (PFM) [2], and discrete element method (DEM) [3]. XFEM is effective for simulating discontinuous problems such as interfaces and crack propagation. Jaskowiec et al. used XFEM for three-dimensional numerical thermo-mechanical modeling of a laminated structure with a very thin inner layer [4]. Kumar et al. employed XFEM for a thermo-mechanical fracture analysis of porous functionally graded cracked plates [5]. PFM is used for simulating structural damage. Badnava et al. used PFM to simulate brittle fracture and thermal cracks in two-dimensional (2D) and three-dimensional (3D) continua [6]. Zhou et al. presented a novel coupled thermo-mechanical PFM for concrete at high temperatures [7]. DEM can reproduce macroscopic behavior comparable to laboratory tests and monitor microscopic variations in the failure process. Sun et al. presented a low-temperature thermo-mechanical coupling modeling framework to simulate frost crack evolution in rock masses using the finite-discrete element method (FDEM) [8]. Although the above methods can solve the thermo-mechanical coupling problem, it is still a challenge to deal with the initiation and propagation of complex multi-cracks.

Silling introduced a novel non-local continuum model known as the peridynamic (PD) model [9]. The PD model has advantages for complex multi-cracks with unknown a priori locations. Unlike the partial differential governing equations used in CCM, the PD model employs integro-differential governing equations, making it suited for simulation of structural fractures. PD naturally simulates crack initiation and propagation without requiring any predefined crack growth criteria. Subsequently, a PMB constitutive model of PD was introduced by Silling [10]. This particular PD model is termed the bond-based peridynamic (BB-PD) model, wherein the Poisson’s ratio remains constrained to a fixed value. Recognizing this limitation, Silling introduced a mathematical construct known as ‘state’ in 2007, thereby presenting two variations: the ordinary state-based peridynamic (OSB-PD) model and the non-ordinary state-based peridynamic (NOSB-PD) model [11]. Notably, the BB-PD model can be viewed as a specialized instance within the broader framework of the state-based peridynamic model [12]. Additionally, PD model demonstrates applicability in addressing thermal conduction and thermal fracture problems. Bobaru and Duangpanya introduced a PD formulation for transient heat conduction in solids with discontinuities [13]. Oterkus et al. derived OSB-PD heat conduction equations [14]. Based on the above works, PD can be used to solve thermo-mechanical problems. Oterkus et al. presented a fully coupled PD thermo-mechanical framework [15]. PD’s inherent ability to simulate crack initiation and propagation has made it practical for engineering applications. Wang et al. developed a thermo-mechanical BB-PD model to simulate thermal cracking processes in concrete exposed to fire scenarios [16]. Zang et al. introduced a fully coupled thermo-mechanical PD model for rock fracturing under blast loading, accounting for initial pore damage [17]. Cheng et al. presented a thermo-mechanical PD model to investigate damage in engineered cementitious composite-concrete bonding specimens at high temperatures [18]. While these studies effectively simulated thermal fracture, selecting the appropriate micro-conductivity remains a challenge. Sun et al. presented a novel computational framework for analyzing thermal fracture in brittle solids by coupling PD and CCM [19]. However, the original PD model defines damage solely based on the number of broken bonds but neglects their spatial distribution, thereby introducing inaccuracies in damage quantification. To address this limitation, we introduce a novel PD damage formulation that considers both the quantity and spatial distribution of broken bonds. As a result, damage in various directions can be captured, enabling the simulation of thermal fracture based on a unified mesh discretization framework.

The structure of the remainder of this paper is organized as follows: Section 2 reviews the fundamental formulations of the BB-PD model and presents a new definition of PD damage within this framework. Section 3 revisits the thermo-mechanical PD model and establishes a framework for integrating the new definition of the PD damage into the model. Section 4 details the finite element spatial discretization, as well as the time discretization, of the proposed framework. Section 5 demonstrates the effectiveness of the proposed model through four examples. Section 6 concludes with remarks summarizing the findings and contributions of this paper.

## 2. A New Definition of PD Damage

In this section, we first review the BB-PD model and the original definition of PD damage. Then, we elucidate the necessity and specific formula of the new definition of PD damage. It should be noted that the new definition of PD damage presented in this paper is applicable to the NOSB-PD model and the OSB-PD model, with the BB-PD model being introduced in detail as a special case.

### 2.1. A Review of the BB-PD Model and PD Damage

The PD model assumes that each material point x has its own neighborhood $H_{\delta }\left(x\right)$ and interacts with points $x^{\prime }$ located in $H_{\delta }\left(x\right)$. The equilibrium equation can be expressed as follows [20]:

(1) $$\begin{array}{c} \int_{H_{\delta }\left(x\right)}f\left(x^{\prime },x\right)dV_{x^{\prime }}+b\left(x\right)=0\;\forall \;x,x^{\prime }\in \Omega \end{array}$$

where $H_{\delta }\left(x\right)$ denotes the neighborhood of x with a horizon of δ; b(x) signifies the external force acting on point x, and $f(x^{\prime },x)$ represents a pairwise force function. A potential constitutive model relating force to relative displacement for linear elasticity and small deformations can be expressed as follows:

(2) $$\begin{array}{c} f\left(x^{\prime },x\right)=\;\frac{1}{2}\left(c(x,|\xi |)+c(x^{\prime },|\xi |)\right)\left(u_{\xi }\left(x^{\prime }\right)-u_{\xi }\left(x\right)\right)e_{\xi } \end{array}$$

where $u_{\xi }\left(x^{\prime }\right)$ and $u_{\xi }\left(x\right)$ represent the projections of the displacement at point x and $x^{\prime }$ onto the bond, respectively; $e_{\xi }$ is the unit vector of bond ξ; c(x,|ξ|); and $(c(x^{\prime },|\xi |))$ denote micro-modulus functions of bond ξ. In this paper, we focus on homogeneous materials (i.e., $c(x,|\xi |)=c(x^{\prime },|\xi |)=c^{0}\left(|\xi |\right)$). The elastic energy density can be expressed as follows [21]:

(3) $$\begin{array}{cc} W(x) & =\frac{1}{4}\int_{H_{\delta }\left(x\right)}c^{0}\left(|\xi |\right){\left(u_{\xi }\left(x^{\prime }\right)-u_{\xi }\left(x\right)\right)}^{2}dV_{x^{\prime }} \end{array}$$

To capture crack initiation and propagation, the PD model requires a bond failure criterion to trigger bond failure. Bond failure can be tracked using a history-dependent function μ, defined as follows:

(4) $$\begin{array}{c} \mu \left(x^{\prime },x,t\right)=\left\{\begin{array}{cc} \;1 & s\;<s_{0}\\ \;0 & \mathrm{otherwise} \end{array}\right. \end{array}$$

where *s* is the bond stretch; *t* is the computational step; and $s_{0}$ denotes the critical bond stretch. The relationship between the bond stretch *s* and displacement can be formulated as follows:

(5) $$\begin{array}{c} s=\frac{|u\left(x^{\prime }\right)-u\left(x\right)+\xi |-|\xi |}{\left|\xi \right|} \end{array}$$

It’s worth noting that the PD model employs a scalar d(x,t) to describe damage at a point x:

(6) $$\begin{array}{c} d\left(x,t\right)=1-\frac{\int_{H_{\delta }\left(x\right)}\mu \left(x^{\prime },x,t\right)dV_{x^{\prime }}}{\int_{H_{\delta }\left(x\right)}dV_{x^{\prime }}} \end{array}$$

### 2.2. A New Definition of PD Damage Based on the Spatial Distribution of the Broken Bonds

**Figure 1:**
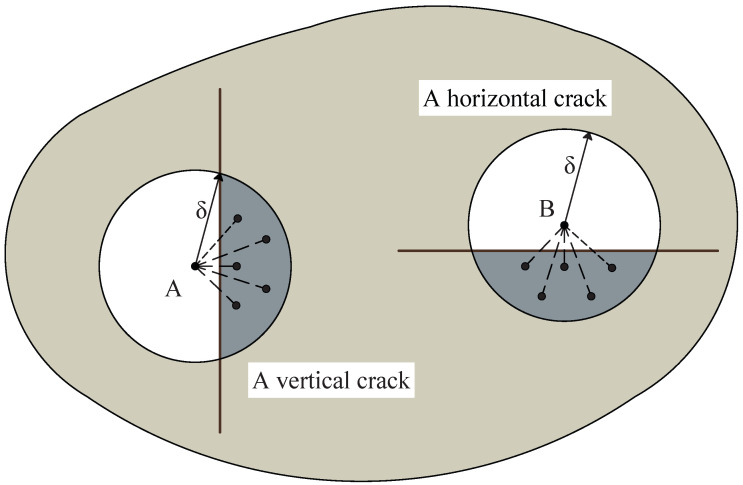
Comparison of the spatial distribution of the broken bonds between points *A* and *B*.

As demonstrated in Equation (6), the original PD damage is calculated based on the proportion of broken bonds to total bonds. While the original PD damage can indeed characterize the degradation of materials, it is difficult to accurately portray the specific bond failure distribution within the neighborhood. To illustrate, consider the scenario depicted in Figure 1, where two differently oriented cracks traverse the neighborhood in a two-dimensional plane. The spatial distribution of the broken bonds differs between the vertical crack and the horizontal crack. However, when Equation (6) is employed to compute the damage values for material points A and B, these two distinct scenarios may have identical damage values (d(A)=d(B)=0.5). It underscores the limitation of the original PD damage in capturing the specific spatial distribution of the broken bonds. On the other hand, the original PD damage establishes a homogenized mapping mechanism between microscopic bond failure and macroscopic damage fields through statistical averaging approaches. However, when addressing multi-physics coupling simulations (e.g., thermo-mechanical or hygro-mechanical interactions), the macroscopic damage field necessitates explicit consideration of anisotropic damage characteristics. This requirement exposes an intrinsic limitation of original PD damage—its inherent isotropy assumption fundamentally restricts the characterization of direction-dependent damage evolution patterns.

To address the aforementioned limitation in the PD damage, it is necessary to implement a new definition of PD damage based on bond distributions. In the new definition, the PD damage can be represented as $\hat{d}$ rather than a scalar to accurately describe damage in various directions:

(7) $$\begin{array}{c} \hat{d}=\left(\begin{array}{ccc} d_{1} & 0 & 0\\ 0 & d_{2} & 0\\ 0 & 0 & d_{3} \end{array}\right) \end{array}$$

where $d_{1}$, $d_{2}$, and $d_{3}$ are components defined in the material coordinate system. For isotropic materials, $d_{1}$, $d_{2}$, and $d_{3}$ represent the damage in the directions of the x, y, and z axes, respectively. And, for anisotropic materials, $d_{1}$, $d_{2}$, and $d_{3}$ represent damage in three main directions. However, due to the symmetry of the PD domain, despite dividing the bond among different directions, for the same PD neighborhood, $d_{1}$, $d_{2}$, and $d_{3}$ are almost equal. Therefore, it remains challenging to distinguish damage among different directions.

**Figure 2:**
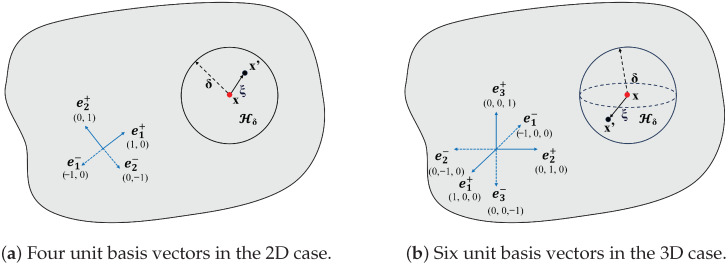
Basis vectors $e_{i}^{*}$ in 2D and 3D cases.

To distinguish $d_{1}$, $d_{2}$, and $d_{3}$, some improvements have been made to present a new definition of the PD damage. As shown in Figure 2, unit basis vectors $e_{i}^{*}\left(*=+,-\right)$ along material principal directions are defined in both 2D and 3D cases. For 2D cases, i=1,2, and for 3D cases, i=1,2,3. To distinguish the positions of each bond ξ in the neighborhood $H_{\delta }$, a scalar function $\nu_{i}^{*}\left(x^{\prime },x\right)$ is defined as follows:

(8) $$\begin{array}{c} \nu_{i}^{*}\left(x^{\prime },x\right)=\left\{\begin{array}{cc} \;1 & e_{i}^{*}\cdot \xi \;>0\\ \;0 & \mathrm{otherwise} \end{array}\right. \end{array}$$

Based on Equations (6) and (8), the following formula can be obtained:

(9) $$\begin{array}{c} d_{i}^{*}\left(x^{\prime },x,t\right)=1-\frac{\int_{H_{\delta }\left(x\right)}\nu_{i}^{*}\left(x^{\prime },x\right)\mu \left(x^{\prime },x,t\right)dV_{x^{\prime }}}{\int_{H_{\delta }\left(x\right)}\nu_{i}^{*}\left(x^{\prime },x\right)dV_{x^{\prime }}} \end{array}$$

where $d_{i}^{*}\left(x^{\prime },x,t\right)$ ($0\leq d_{i}^{*}\left(x^{\prime },x,t\right)\leq 1$) denotes damage in each direction *i*, ∗. For 2D cases, i=1,2, and for 3D cases, i=1,2,3.

Having computed the directional damage components $d_{i}^{*}\left(x,t\right)$ for each hemisphere (∗=+,−), the next step is to assemble them into a damage tensor $\hat{d}\left(x,t\right)$. A critical choice is the aggregation rule for the two hemispheres along each principal direction *i*.

**Physical and Mathematical Justification for the Maximum Rule**

The tensor components are constructed using the *maximum* of the two hemispherical damage values:

(10) $$d_{i}=\max\left\{d_{i}^{+},d_{i}^{-}\right\}.$$

This choice is motivated by both physical reasoning and mathematical pragmatism.

*Physical Motivation:* In brittle fracture, the macroscopic material degradation (e.g., loss of stiffness or thermal conductivity) along a given direction is dominantly governed by the most severe local damage within that directional neighborhood. A crack, which is a localized plane of broken bonds, will severely degrade properties in its normal direction regardless of the state of the opposite hemisphere. The maximum rule adopts a conservative engineering perspective by ensuring that the damage tensor reflects the envelope of directional damage severity, which is crucial for accurately driving coupled processes like anisotropic thermal conductivity reduction (see Equation (21)).

*Mathematical Motivation:* The goal is to map a discrete set of bond failures to a continuous, symmetric second-order tensor suitable for constitutive modeling. Alternative rules, such as taking the average ($(d_{i}^{+}+d_{i}^{-})/2$), can smooth out localized damage. For instance, if severe damage exists in one hemisphere ($d_{i}^{+}\approx 1$) while the opposite is nearly intact ($d_{i}^{-}\approx 0$), the average (≈0.5) would significantly underestimate the true degradation along that direction. The maximum rule preserves the monotonicity between microscopic bond failure and the macroscopic damage measure and naturally yields a symmetric, positive semi-definite damage tensor.

*Behavior under Symmetric and Asymmetric Damage:*

- **Symmetric Damage:** If damage is diffuse and approximately equal in both hemispheres ($d_{i}^{+}\approx d_{i}^{-}$), then $\max\left\{d_{i}^{+},d_{i}^{-}\right\}\approx d_{i}^{+}\approx d_{i}^{-}$. The rule effectively reduces to a representative average for that direction.
- **Asymmetric Damage:** This is the typical case for a localized crack. The rule selects the hemisphere with the more severe damage as the representative value for direction *i*. Information about the less damaged hemisphere is not entirely lost, as it may influence the damage components in other directions. The full tensor $\hat{d}$, through the differences among its diagonal components, still captures the overall anisotropic damage pattern.

Therefore, the maximum rule provides a robust, conservative, and physically interpretable method for constructing a damage tensor from directional bond failure statistics.

For 2D cases, the new definition of the PD damage can be written as follows:

(11) $$\begin{array}{c} \hat{d}=\left(\begin{array}{cc} d_{1} & 0\\ 0 & d_{2} \end{array}\right)=\left(\begin{array}{cc} max\{d_{1}^{+},d_{1}^{-}\} & 0\\ 0 & max\{d_{2}^{+},d_{2}^{-}\} \end{array}\right) \end{array}$$

This means that the maximum vector should be selected among all possible damage vectors. Similarly, for 3D cases, the new definition of the PD damage can be written as follows:

(12) $$\begin{array}{c} \hat{d}=\left(\begin{array}{ccc} d_{1} & 0 & 0\\ 0 & d_{2} & 0\\ 0 & 0 & d_{3} \end{array}\right)=\left(\begin{array}{ccc} max\{d_{1}^{+},d_{1}^{-}\} & 0 & 0\\ 0 & max\{d_{2}^{+},d_{2}^{-}\} & 0\\ 0 & 0 & max\{d_{3}^{+},d_{3}^{-}\} \end{array}\right) \end{array}$$

**Figure 3:**
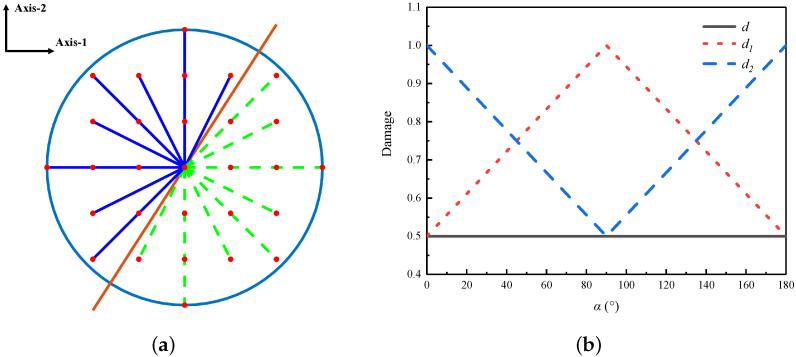
Comparison of damage at different crack angles. (**a**) Crack path with an angle of α to the principal axes of material. (**b**) The classical damage and the redefined damage.

As shown in Figure 1, for point *A*, the new definition of the PD damage $\hat{d}=\left(\begin{array}{cc} 1 & 0\\ 0 & 0.5 \end{array}\right)$, whereas for point *B*, $\hat{d}=\left(\begin{array}{cc} 0.5 & 0\\ 0 & 1 \end{array}\right)$. This means that the new definition of the PD damage varies depending on different bond failure distributions within the PD domain. In order to further clarify the effectiveness of the new definition of the PD damage in characterizing cracks, as depicted in Figure 3a, consider a crack surface that intersects the horizontal plane. Assume that all bonds crossing the crack surface are broken. The angle between this crack surface and the principal axes of the material is denoted as α. Broken bonds are indicated by green dashed lines, while intact bonds are represented by blue solid lines. Furthermore, as illustrated in Figure 3b, the red line represents $d_{1}$, the blue line represents $d_{2}$, and the black line represents the original PD damage. As the angle α changes, the new definition of the PD damage also varies accordingly. When the angle is $0^{\circ }$, $d_{2}$ is the maximum and $d_{1}$ is the minimum. When the angle is $90^{\circ }$, $d_{1}$ is the maximum and $d_{2}$ is the minimum.

### 2.3. Generalization to State-Based Peridynamic Models

The novel damage tensor $\hat{d}$ proposed in Equations (11) and (12) is formulated within the BB-PD framework for clarity. However, its core concept—quantifying damage by counting broken bonds in different spatial directions—is general and can be directly extended to state-based peridynamics, including both OSB-PD and NOSB-PD models.

In state-based peridynamics, the pairwise force function is replaced by a more general force state, but the geometric concept of a “bond” as a connection between two material points persists. Therefore, the bond-failure indicator $\mu (x^{\prime },x,t)$ can still be defined according to a suitable failure criterion appropriate for the state-based model. Common choices include:

- A critical stretch criterion analogous to Equation (4) for certain material models.
- An energy-based criterion where a bond breaks when its contribution to the strain energy density reaches a critical fracture energy $G_{c}$.
- A stress- or strain-invariant-based criterion, especially in the NOSB-PD model, where bonds associated with a point are considered broken when a local stress or strain measure exceeds the material strength.

Once $\mu (x^{\prime },x,t)$ is defined, the subsequent formulas for calculating the directional damage components $d_{i}^{*}\left(x,t\right)$ (Equation (9)) and assembling the damage tensor $\hat{d}$ (Equations (11) and (12)) remain identical to those presented in the bond-based formulation. The key step is the directional counting of broken bonds via the half-space indicator $\nu_{i}^{*}\left(x^{\prime },x\right)$ (Equation (8)), which is purely geometric and independent of the constitutive law.

Thus, the proposed damage tensor provides a unified measure for anisotropic damage evolution that can be coupled with multi-physics processes (e.g., anisotropic thermal conductivity reduction as in Equation (21) within both BB-PD and state-based peridynamic frameworks.

The subsequent sections introduce the applications and benefits of the new definition of PD damage in modeling thermal fracture.

## 3. A New Definition of PD Damage for Modeling Thermal Fracture

### 3.1. An Improved Thermo-Mechanical PD Model

The PD equilibrium equation with temperature can be expressed as follows:

(13) $$\begin{array}{c} \int_{H_{\delta }\left(x\right)}f\left(x^{\prime },x,T\right)dV_{x^{\prime }}+b\left(x\right)=0\;\forall \;x,x^{\prime }\in \Omega \end{array}$$

The bond force can be divided into two parts:

(14) $$\begin{array}{c} f\left(x^{\prime },x,\hat{T}\right)=\hat{f}\left(x^{\prime },x,\hat{T}\right)-\hat{f}\left(x,x^{\prime },\hat{T}\right) \end{array}$$

where $\hat{f}\left(x^{\prime },x,\hat{T}\right)$ and $\hat{f}\left(x,x^{\prime },\hat{T}\right)$ are the bond forces of point $x^{\prime }$ over point x and x over point $x^{\prime }$; $\hat{T}$ is the temperature variation of the bond. A possible constitutive equation can be written as follows [22]:

(15) $$\begin{array}{c} \hat{f}\left(x^{\prime },x,\hat{T}\right)=\frac{1}{2}c\left(x,|\xi |\right)\left(u_{\xi }\left(x^{\prime }\right)-u_{\xi }\left(x\right)-a\left(x\right)\hat{T}\left(x^{\prime },\xi \right)\right)e_{\xi } \end{array}$$

where a(x) denotes micro-expansivity at point x; for homogeneous materials, $a\left(x\right)=a\left(x^{\prime }\right)=a^{0}$. Substitute Equation (15) into Equation (14):

(16) $$\begin{array}{cc} f(x^{\prime },x,\hat{T}) & =\;\frac{1}{2}\left(c(x,|\xi |)+c(x^{\prime },|\xi |)\right)\left(u_{\xi }\left(x^{\prime }\right)-u_{\xi }\left(x\right)\right)e_{\xi }\\ & -\frac{1}{2}\left(b(x,|\xi |)+b(x^{\prime },|\xi |)\right)\hat{T}\left(x,\xi \right)e_{\xi } \end{array}$$

where b(x,|ξ|) is the thermal modulus, written as follows:

(17) $$\begin{array}{c} b\left(x,\xi \right)=a\left(x\right)c\left(x,|\xi |\right)=a^{0}c^{0}\left(|\xi |\right) \end{array}$$

In this paper, we focus on homogeneous materials. (i.e., $b(x,|\xi |)=b(x^{\prime },|\xi |)=b^{0}\left(|\xi |\right)$).

Consider a uniform and isotropic solid containing a heat source and undergoing heat exchange with its surrounding medium. We study the distribution and variations of temperature within the solid. This analysis is grounded in the principles of the energy conservation equation:

(18) $$\begin{array}{c} \frac{d}{dt}\underset{R}{\;\int \int \int \;}c\rho TdV=-\underset{R}{\;\int \int \int \;}\nabla \cdot JdV+\underset{R}{\;\int \int \int \;}QdV. \end{array}$$

where *c* is the specific heat capacity; ρ is the density; T is the temperature; J is the heat flux; *t* is time, Q is the heat source. The above equation can be simplified as follows:

(19) $$\begin{array}{c} \rho c\dot{T}+\nabla \cdot J=Q \end{array}$$

According to the Fourier law,

(20) $$\begin{array}{c} J=-k\nabla T \end{array}$$

where k is the thermal conductivity.

### 3.2. Anisotropic Thermal Conductivity for Modeling Thermal Fracture

**Figure 4:**
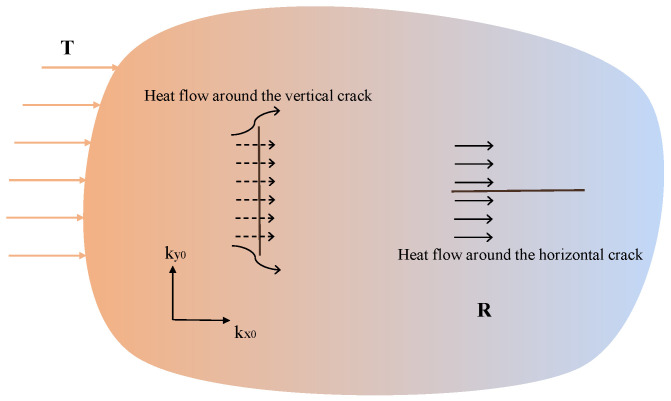
Heat flow around the vertical crack and horizontal crack.

According to Section 3.1, in the thermo-mechanical model, the temperature field and deformation field can interact with each other. Changes in temperature, whether increasing or decreasing, affect the material’s thermal deformation. The bond failure can be calculated through deformation fields. Progressive accumulation of microscopic bond failures induces macroscopic damage, which can locally characterize the degradation of thermal conductivity. As shown in Figure 4, a fixed temperature T is applied to the left boundary of the solid. When considering heat flow through both horizontal and vertical cracks, it is observed that the vertical crack significantly hinders heat flow, while the horizontal crack has a weaker impact. This demonstrates that cracks oriented in different directions can hinder heat flow to varying degrees. Therefore, the reduction in thermal conductivity cannot solely be attributed to the original PD damage, as described by Equation (6), and must also consider the direction and extent of cracking.

A suitable way to define thermal conductivity k based on PD damage is as follows:

(21) $$\begin{array}{c} k=\left(I-\hat{d}\right)k_{0} \end{array}$$

where $k_{0}$ is the reference thermal conductivity; I is the unit matrix; $\hat{d}$ is defined in Equations (11) and (12). This formula reflects the anisotropic effect of damage on thermal conductivity, with different degrees of degradation in different directions.

## 4. Numerical Algorithm

### 4.1. A Unified Finite Element Discretization for Thermo-Mechanical Crack Propagation

**Figure 5:**
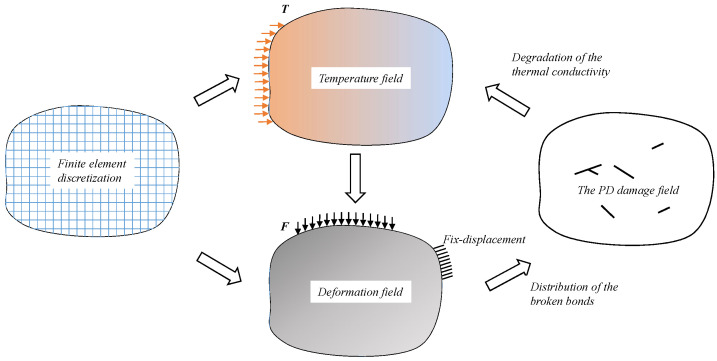
The computation framework of the unified finite element discretization for thermo-mechanical crack propagation.

In a previous study [19], temperature fields were computed using a local model based on the finite element discretization, while deformation fields with discontinuities were computed using a PD model based on an element-free discretization. In this paper, we unify the discretization of both model through a shared mesh system. Figure 5 illustrates the proposed computational framework, where finite element discretization of the computational domain is implemented during the initialization phase. The computational procedure initiates with the computation of the temperature field. Sequentially, the temperature field is incorporated into the PD model to obtain the deformation field. The deformation field is used to determine bond failures. Progressive accumulation of bond failures induces micro-crack nucleation. Micro-cracks coalesce through damaged bands to form macro-cracks. The PD damage defined in this paper enables characterization of degradation in thermal conductivity, affecting the distribution of the temperature field.

### 4.2. Time and Spatial Discretization

The time discretization of the temperature field is as follows:

(22) $$T(n+\theta \Delta t)=\left(1-\theta \right)T_{n}+\theta T_{n+1}$$

(23) $$\dot{T}\left(n+\theta \Delta t\right)=(T_{n+1}-T_{n})/\Delta t$$

where Δt is the time increment; θ is the integration parameter; different values of θ correspond to different differential formulas. $T_{n+1}$ and $T_{n}$ are temperatures at time step n+1 and *n*.

The governing equation for temperature field computation can be expressed in the following canonical finite element form:

(24) $$\begin{array}{c} C\dot{T}+KT=P \end{array}$$

where C is the heat capacity matrix; K is the heat conduction matrix; T is the temperature vector; P is the temperature load vector; $\dot{T}$ is the derivative vector of node temperature with respect to time.

The heat conduction matrix K, the heat capacity matrix C, and the temperature load vector P can be written as follows:

(25) $$\begin{array}{cc} K & =\sum_{i=1}^{n}\int_{V_{i}}{\left(HN_{i}\left(x\right)R_{i}\right)}^{T}k\left(HN_{i}\left(x\right)R_{i}\right)dV_{x}\\ & +\sum_{i=1}^{n}\int_{S_{i}^{3}}h{\left(N_{i}\left(x\right)R_{i}\right)}^{T}\left(N_{i}\left(x\right)R_{i}\right)dS_{x}^{3}\\ C & =\sum_{i=1}^{n}\int_{V_{i}}\rho c{\left(N_{i}\left(x\right)R_{i}\right)}^{T}\left(N_{i}\left(x\right)R_{i}\right)dV_{x}\\ P & =\sum_{i=1}^{n}\int_{V_{i}}\rho Q\left(x\right){\left(N_{i}\left(x\right)R_{i}\right)}^{T}dV_{x}\\ & -\sum_{i=1}^{n}\int_{S_{i}^{2}}q\left(x\right){\left(N_{i}\left(x\right)R_{i}\right)}^{T}dS_{x}^{2}\\ & +\sum_{i=1}^{n}\int_{S_{i}^{3}}h\phi_{a}{\left(N_{i}\left(x\right)R_{i}\right)}^{T}dS_{x}^{3} \end{array}$$

where *n* is the number of total finite elements; *h* is the heat convection coefficient corresponding to the boundary $S^{3}$; $\phi_{a}$ is the temperature on boundary $S^{3}$; q is the heat flux density corresponding to the boundary $S^{2}$; N denotes the matrix of shape function; H denotes the matrix of differential operators; QQ is the heat source.

The finite element spatial discretization of PD model can be written as the following formula [23]:

(26) $$\begin{array}{c} \hat{K}d=F \end{array}$$

where $\hat{K}$ is the total stiffness matrix; d is the displacement vector; F is the external load force vector.

The total stiffness matrix $\hat{K}$ and the external load force vector F can be written as follows:

(27) $$\begin{array}{cc} \hat{K} & =\frac{1}{2}\sum_{i=1}^{n}\sum_{j=1}^{\hat{h}\left(x\right)}\int_{V_{i}}\int_{V_{x}^{j}}c^{0}\left(|\xi |\right){\left(N_{j}\left(x^{\prime }\right)R_{j}-N_{i}\left(x\right)R_{i}\right)}^{T}\frac{\xi ⊗\xi }{{\left|\xi \right|}^{2}}\left(N_{j}\left(x^{\prime }\right)R_{j}-N_{i}\left(x\right)R_{i}\right)dV_{x^{\prime }}dV_{x}\\ F & =\sum_{i=1}^{n}\int_{V_{i}}{\left(N_{i}\left(x\right)R_{i}\right)}^{T}b\left(x\right)dV_{x}+\sum_{i=1}^{n}\int_{S_{i}}{\left(N_{i}\left(x\right)R_{i}\right)}^{T}\bar{F}\left(x\right)dS_{x}\\ \; & +\frac{1}{2}\sum_{i=1}^{n}\sum_{j=1}^{\hat{h}\left(x\right)}\int_{V_{i}}\int_{V_{x}^{j}}b^{0}\left(|\xi |\right){\left(N_{j}\left(x^{\prime }\right)R_{j}-N_{i}\left(x\right)R_{i}\right)}^{T}\frac{\xi }{\left|\xi \right|}TdV_{x^{\prime }}dV_{x} \end{array}$$

where $\hat{h}\left(x\right)$ is the amount of relative elements of point x; b is the body force.

### 4.3. Flowchart of the Proposed Numerical Algorithm

**Figure 6:**
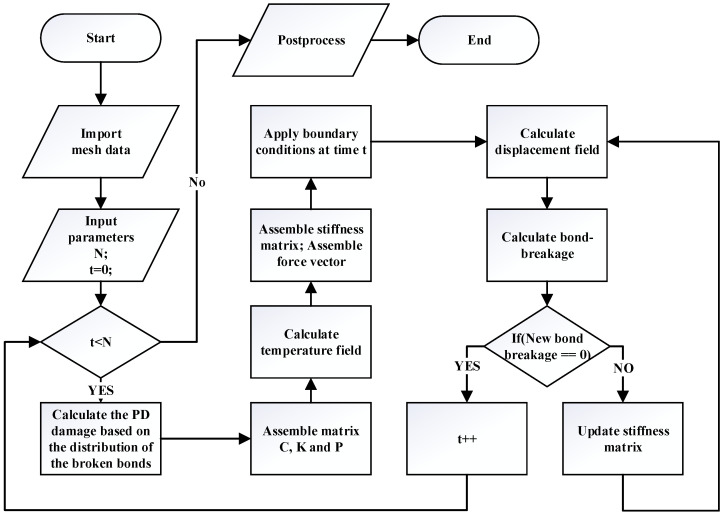
Flowchart of the numerical algorithm.

As depicted in the flowchart in Figure 6, to address thermal fracture problems based on a shared mesh system between temperature and deformation computation, the temperature field and deformation field are computed independently. At the beginning of the algorithm, the geometric model only needs to be discretized once. Subsequently, for each time step, the new definition of the PD damage is computed to characterize the degradation of the thermal conductivity. We solve the linear Equation (24) to obtain the temperature field, which is subsequently utilized to compute the equilibrium Equation (26), incorporating temperature terms and obtain displacement field. The displacement field allows us to identify bond failures. Progressive accumulation of bond failures induces micro-crack nucleation. For quasi-static problems, if no new bond failure is detected, we proceed to the calculation for the subsequent time step, continuing this process until the end of the computation.

## 5. Numerical Examples

### 5.1. Thermal Deformation Without Damage

**Figure 7:**
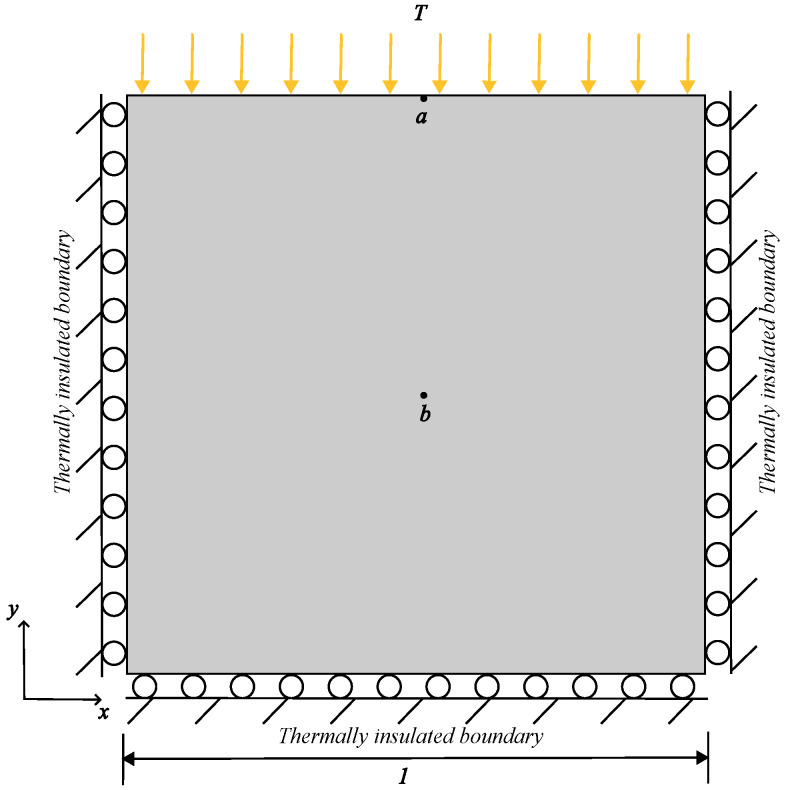
Sketch of the square plate (unit: m).

To validate the efficacy of the proposed model, we consider a scenario involving thermal deformation without damage. The condition of plane strain is adopted for this example. Timoshenko et al. [24] analyzed a square plate with three edges thermally insulated and mechanically restrained against normal displacement. As illustrated in Figure 7, the top edge of the plate is subject to a Dirichlet boundary condition with a temperature T=1 °C, while the initial temperature $T_{0}$ of the whole plate is 0 °C. The material properties are detailed in Table 1.

Timoshenko et al. [24] and Carslaw and Jaeger [25] derived the analytical solution for this problem as follows:

(28) $$\begin{array}{c} T\left(y,t\right)=1-\frac{4}{\pi }\sum_{n=0}^{\infty }\frac{{\left(-1\right)}^{n}}{2n+1}\exp\left(-\frac{\left(2n+1\right)\pi^{2}kt}{4L^{2}}\right)cos\left(\frac{\left(2n+1\right)\pi y}{2L}\right) \end{array}$$

and

(29) $$\begin{array}{c} u_{y}\left(y,t\right)=\frac{\left(1+\nu \right)}{\left(1-\nu \right)}\alpha \int_{0}^{y}T\left(y,t\right)dy \end{array}$$

**Figure 8:**
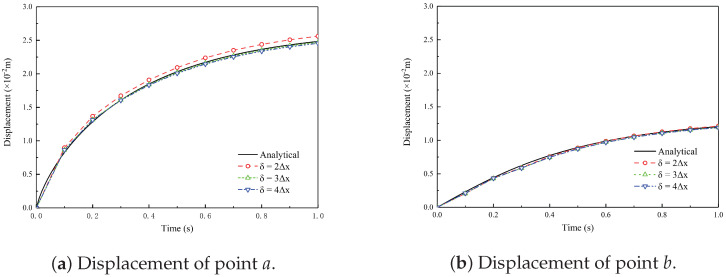
Comparison of vertical displacement of points *a* and *b* between analytical and PD solutions with different horizons.

In this numerical example, the PD micromodulus coefficient is assumed to be an exponential function: $c^{0}\left(∥\xi ∥\right)=\tau^{0}e^{-∥\xi ∥/l}$, where $\tau^{0}$ is a constant coefficient that is calculated according to the given Poisson’s ratio and Young’s modulus, and *l* is a characteristic length. In this example, l=δ/3. The model is discretized into 10,000 uniform quadrilateral finite elements with dimensions of 10×10 mm. Figure 8 shows the vertical displacement of points *a* and *b* with different horizons, i.e., δ=2Δx,3Δx,4Δx, where Δx is the element size. By comparing with the analytical solution, the thermo-mechanical PD model is validated. In order to improve computational efficiency and obtain accurate calculation results, δ=3Δx is a suitable choice of horizon.

### 5.2. Heat Flow in a Plate with Two Thermal Insulation Cracks

**Figure 9:**
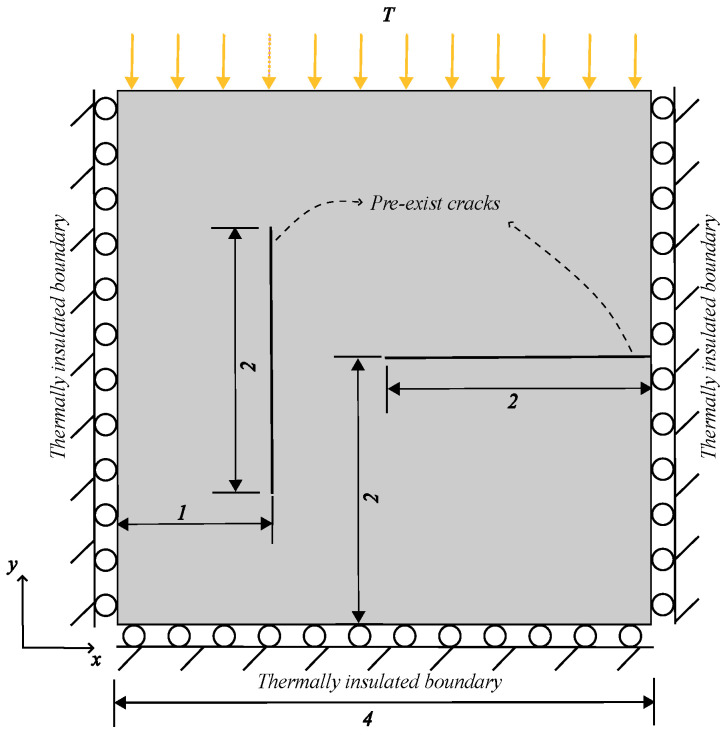
Sketch of the plate with thermal insulation cracks (unit: m).

To validate the proposed new definition of the PD damage, heat flow in a plate containing thermal insulation cracks is considered. The geometry configuration and boundary conditions are shown in Figure 9. There are two pre-existing thermal insulation cracks, one oriented horizontally and the other vertically. The top edge of the plate is subjected to a Dirichlet boundary condition with a temperature of T=100 °C. The initial temperature $T_{0}$ of the whole plate is 0 °C. The material properties are shown in Table 1. The model is discretized into 10,000 uniform quadrilateral finite elements with dimensions of 40×40 mm. The horizon is δ=3Δx.

For comparison purposes, a classical approach to model thermal insulation cracks adopts an isotropic degradation of thermal conductivity based on a scalar damage variable. In the previous model, the thermal conductivity is expressed as:

(30) $$\begin{array}{c} k={\left(1-\bar{d}\right)}^{2}k_{0} \end{array}$$

where $\bar{d}$ is thresholded scalar damage, defined as:

(31) $$\begin{array}{c} \bar{d}=\left\{\begin{array}{cc} \;0 & \mathrm{if}\;d⩽c_{1}\\ \frac{d-c_{1}}{c_{2}-c_{1}} & \mathrm{if}\;c_{1}<d⩽c_{2}\\ \;1 & \mathrm{if}\;d>c_{2} \end{array}\right.. \end{array}$$

Here, *d* is the original scalar damage defined by Equation (6), and $c_{1}$ and $c_{2}$ are two threshold values. This model assumes that the influence of cracks on thermal conductivity is the same in all directions (isotropic).

To account for directional effects, we propose an anisotropic thermal conductivity model based on the new damage tensor $\hat{d}$. A suitable way to define thermal conductivity k has been given in Equation (21).

**Figure 10:**
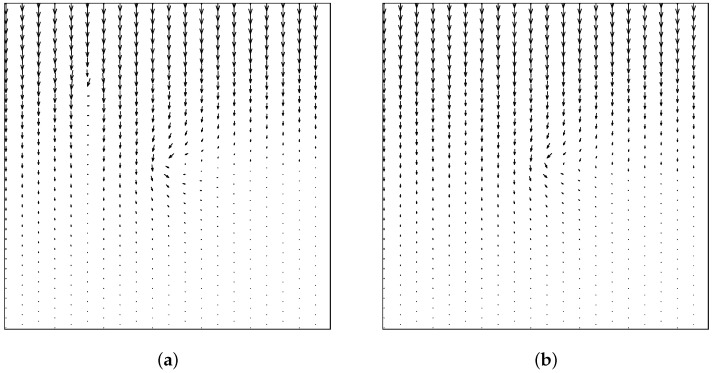
Comparison of heat flux at t=1 s between two models. (**a**) Heat flux calculated using the classical model (isotropic) for thermal insulation cracks. (**b**) Heat flux calculated using the proposed model (anisotropic) for thermal insulation cracks.

**Figure 11:**
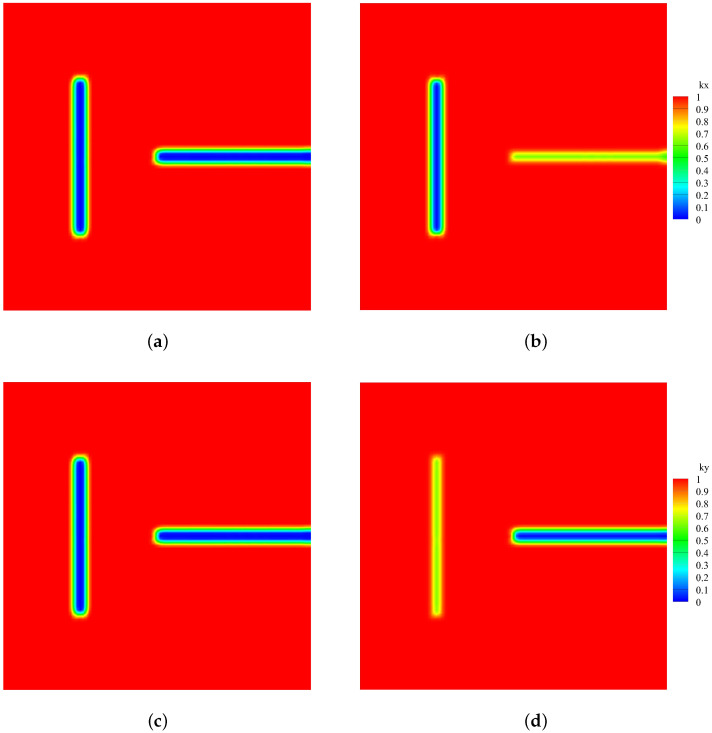
Comparison of thermal conductivity between two models. (**a**) $k_{x}$ calculated using the classical model for thermal insulation cracks. (**b**) $k_{x}$ calculated using the proposed model for thermal insulation cracks. (**c**) $k_{y}$ calculated using the classical model for thermal insulation cracks. (**d**) $k_{y}$ calculated using the proposed model for thermal insulation cracks.

**Figure 12:**
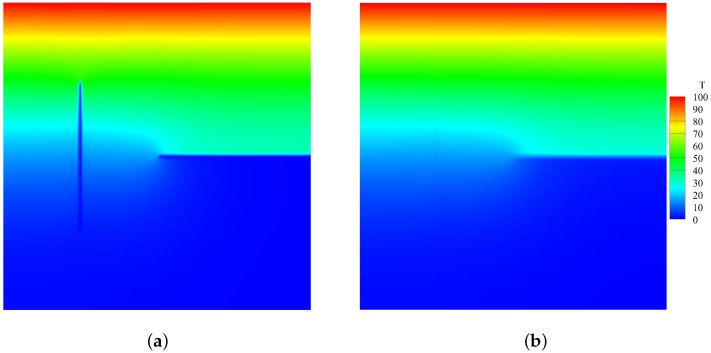
Comparison of temperature field contours between two models (unit: °C). (**a**) Temperature field contour calculated using the classical model for thermal insulation cracks. (**b**) Temperature field contour calculated using the proposed model for thermal insulation cracks.

The calculation results based on Equations (30) and (31) and the proposed model based on Equation (21) for thermal insulation cracks are compared. Figure 10 shows the heat flux calculation results between the classical and the proposed model for thermal insulation cracks. Figure 11 shows the comparison of thermal conductivity $k_{x}$ and $k_{y}$ between the two models. Notably, the classical model calculates thermal conductivity based on a scalar damage value, resulting in equal thermal conductivity in both directions. In contrast, the new definition of the PD damage captures directional differences in thermal conductivity. Specifically, the pre-existing crack in the y-direction significantly affects the thermal conductivity in the x-direction but has minimal impact on the thermal conductivity in the y-direction. Conversely, the effect of cracks in the x-direction on thermal conductivity is opposite. Figure 12 compares the temperature field contours between the two models, further demonstrating the necessity and advantages of the proposed model.

### 5.3. Thermal Fracture of a Cruciform Plate with a Corner Crack

**Figure 13:**
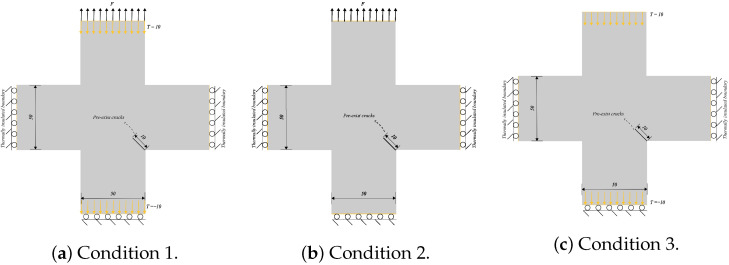
Sketch of the cruciform plate with a corner crack (unit: mm).

This example is a quasi-static crack propagation problem under thermo-mechanical conditions. Figure 13 depicts a cruciform plate with a corner crack, where the crack length is 10 mm and forms a $45^{\circ }$ angle with the vertical axis. The numerical computations are conducted under the assumption of plane stress. We consider crack propagation paths under three distinct mechanical and thermal boundary conditions. In all three conditions, the initial temperature is set to 0 °C, and the three edges of the plate are mechanically restrained against normal displacement. The material properties are shown in Table 2, while the boundary conditions for Figure 13 are detailed in Table 3. To accelerate the calculations, we introduce the PD domain only in the vicinity of the corner crack and employ the CCM model in the remaining domain. The PD domain is discretized into quadrilateral finite element meshes with dimensions of 1×1 mm, while the CCM domain is discretized into finite element meshes with dimensions of 5×5 mm. The PD horizon is set to δ=3Δx.

**Figure 14:**
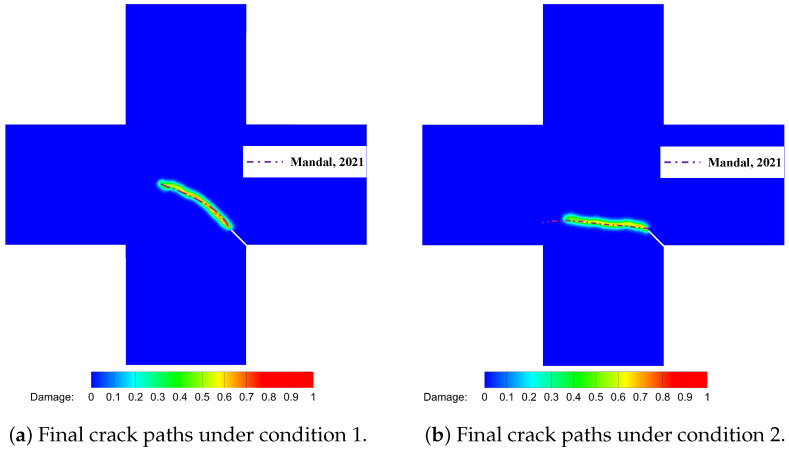
Comparison of crack paths under conditions 1 and 2 between Mandal et al. [26] (represented by purple dashed lines) and the present model (represented by colored contour).

**Figure 15:**
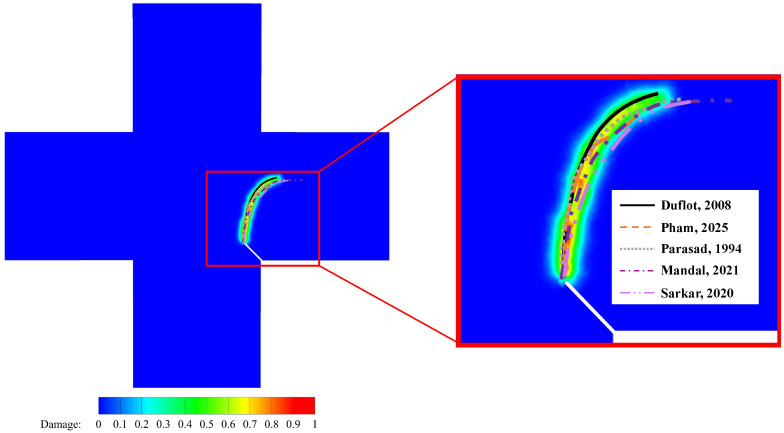
Comparison of crack paths under condition 3 between Mandal et al. [26], Duflot et al. [27], Pham et al. [28], Prasad et al. [29], Sarkar et al. [30], and the present model (represented by colored contour).

**Figure 16:**
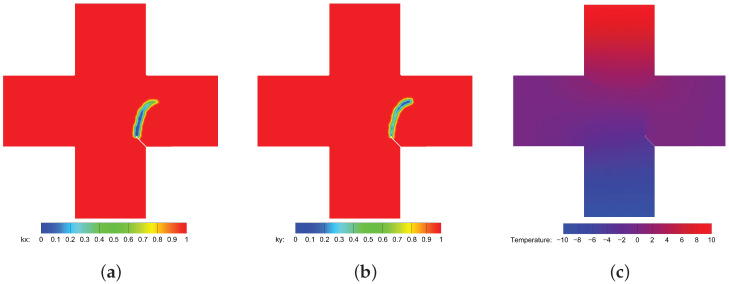
Calculation results of thermal conductivity and temperature field of the proposed model. (**a**) Thermal conductivity $k_{x}$. (**b**) Thermal conductivity $k_{y}$. (**c**) Temperature field contour.

Figure 14 displays the final crack paths under conditions 1 and 2 based on the present model (represented by colored contours), compared with those predicted using PFM proposed by Mandal et al. [26] (represented by purple dashed lines), showing good agreement between the two models. For condition 3, as illustrated in Figure 15, we compare the crack paths obtained from various methods: PFM by Mandal et al. [26], XFEM by Duflot et al. [27], the adaptive mesh refinement (AMR) method by Pham et al. [28], the boundary element method (BEM) by Prasad et al. [29], the gradient-enhanced damage method by Sarkar et al. [30], and the present method (represented by colored contours). As shown, the crack paths predicted using these methods are in good agreement, validating the capability of the present model for simulating thermo-mechanically coupled crack propagation. The new definition of PD damage, which can distinguish damage in two directions, enables a more accurate description of the effect of thermal insulation cracks on thermal conductivity. Figure 16a,b show the degradation of thermal conductivity $k_{x}$ and $k_{y}$ due to the thermal insulation crack. Figure 16c shows the temperature field.

### 5.4. Thermal Shock Fractures in Ceramics

**Figure 17:**
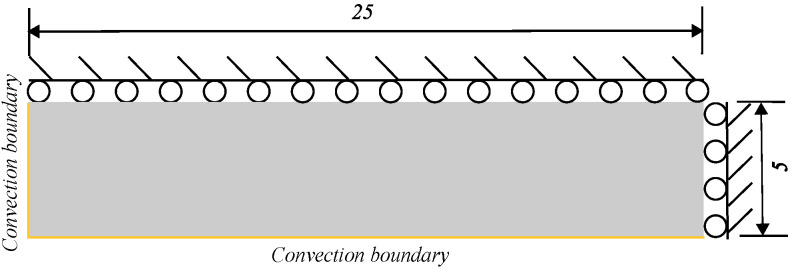
Sketch of thin ceramics plate (unit: mm).

**Figure 18:**
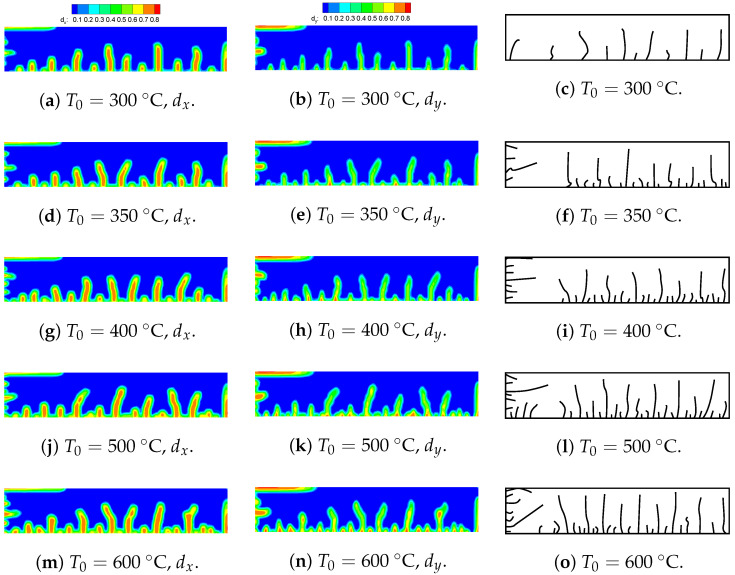
Final crack patterns obtained via the proposed model and the experiment [31]. Reprinted with permission from [31]. Copyright 2012, Elsevier. Permission conveyed through Copyright Clearance Center, Inc.

**Figure 19:**
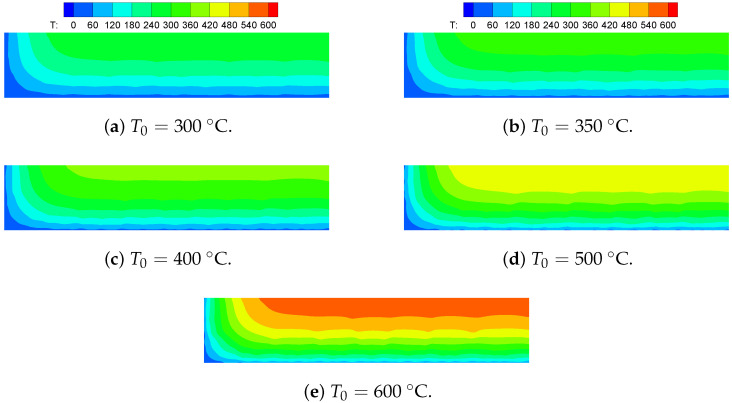
Final temperature field contour obtained via the proposed model.

**Figure 20:**
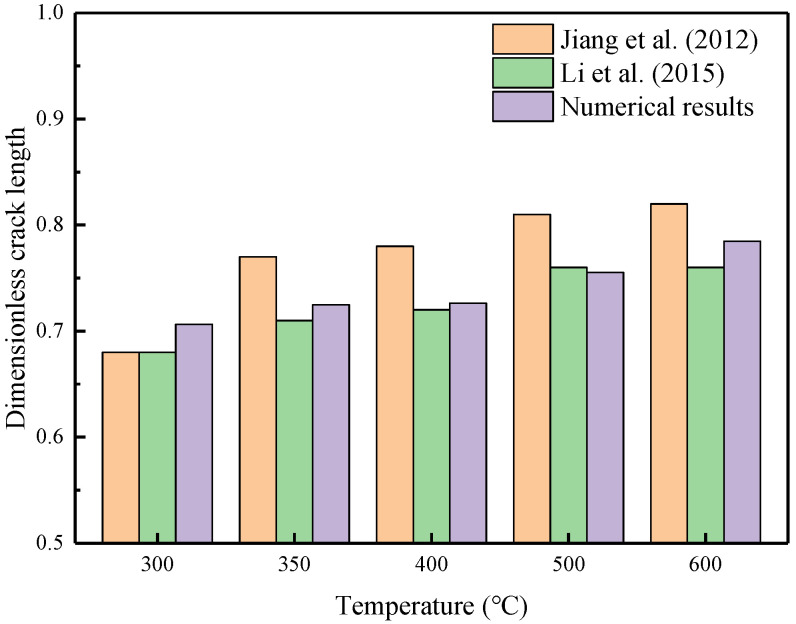
Comparisons between the numerical and experimental results [31,32].

Ceramic materials exhibit excellent mechanical properties at high temperatures, but they may break when subjected to sudden temperature changes. Consequently, thermal shock resistance is a crucial metric for assessing the suitability of ceramic materials for high-temperature engineering applications. The quenching test of ceramics is a widely adopted method to investigate the failure mechanisms associated with thermal shock-induced crack patterns. In the previous experiments, thin specimens measuring 50×10×1 mm were heated to temperature $T_{0}$ and then rapidly quenched in a water bath maintained at T=20 °C [31]. Due to the central symmetry of the boundary conditions and geometric model, only a quarter of the numerical model, with dimensions of 25×10 mm, needs to be simulated. Figure 17 presents a simplified two-dimensional numerical simulation sketch, illustrating the geometry and boundary conditions of the computational domain. The upper and right edges are constrained, while convection boundaries are applied to the left and bottom edges. The numerical computations are based on the plane stress assumption. Table 4 lists the material properties reported in [19,32]. A uniform finite element discretization with a size of 0.1×0.1 mm is employed to calculate both the temperature and displacement fields. The PD horizon is set to δ=3Δx. Figure 18 compares the final crack patterns obtained through numerical simulations and experiments [32]. Both methods demonstrate crack propagation under thermal shock conditions. The initial temperature $T_{0}$ and convective heat transform coefficient $h_{s}$ of the ceramic plates influence the final crack patterns. The left and middle columns of the figure show the new definition of the PD damage $\hat{d}$ proposed in this paper for different $T_{0}$ values. The right column displays the final crack patterns obtained from experiments [32]. Figure 19 presents the final temperature field contours obtained through numerical simulations for various initial temperatures. To compare numerical simulations with experiments, the dimensionless crack length is selected as a reference index. The dimensionless crack length is defined as the ratio of the crack length to the total height (5 mm). Cracks with a dimensionless crack length greater than 0.6 are classified as long cracks. Figure 20 compares the numerical results from the proposed model with experimental results. The crack length in numerical simulations is slightly shorter than that observed in experiments. This discrepancy may be attributed to unpredictable manufacturing defects or material heterogeneities in the ceramic specimens used in the experiments.

## 6. Conclusions

A new definition of PD damage has been developed to model thermal fractures. A comprehensive solution framework has been proposed to compute both the temperature and displacement fields. Specifically, the temperature field is calculated via CCM, whereas the displacement field is computed via PD. To ensure accuracy, unified finite element discretization is employed for both methods. To elucidate the impact of thermal cracks or defects on the temperature field, the insulation crack is modeled by reducing thermal conductivity through the new definition of the PD damage, which indicates the influence of various bond distributions on thermal conductivity.

The proposed thermo-mechanical model utilizes finite element discretization. The shared mesh system for both temperature and displacement computation eliminates the need for remeshing, making it highly efficient and convenient for engineering applications. Furthermore, the new definition of PD damage differs from the original definition. The original definition can only represent the number of bond failures within the domain and cannot depict specific bond failure distributions. In contrast, the new definition of PD damage can characterize damage in different directions. Notably, the model presented in this paper is applicable not only to isotropic materials but also to anisotropic materials. For isotropic materials, the new PD damage is defined along the three coordinate axis directions, whereas for anisotropic materials, it is defined along the material’s three principal directions. Subsequent studies will provide a discussion.

## Figures and Tables

**Table 1:** Material properties of the square plate.

Parameter	Value	Unit
Young’s modulus *E*	1	Pa
Poisson’s ratio ν	0.25	–
Density ρ	0.0	kg/m^3^
Thermal conductivity *k*	1.0	J/(s· m· K)
Specific heat capacity *c*	1.0	J/(kg · K)
Thermal expansion coefficient α	0.016	1/K

Note: The density ρ=0.0 kg/m^3^ is used to enforce quasi-static mechanical equilibrium. A nominal value of ρ=1.0 kg/m^3^ is employed in the transient heat conduction equation to maintain the correct thermal timescale.

**Table 2:** Material properties of the cruciform plate.

Parameter	Value	Unit
Young’s modulus *E*	2.184 $\times 10^{5}$	Pa
Poisson’s ratio ν	0.33	–
Thermal conductivity *k*	1.0	J/(s· m· K)
Specific heat capacity *c*	1.0	J/(kg · K)
Thermal expansion coefficient α	6.0 $\times 10^{-4}$	1/K
Fracture energy *G*	2.0 $\times 10^{-4}$	N/m

**Table 3:** Three boundary conditions of the cruciform plate.

	Temperature (°C)		
BC	Upper	Bottom	Displacement of the Top Edge (mm)
1	10	−10	5.0 $\times 10^{-4}$
2	0	0	5.0 $\times 10^{-4}$
3	10	−10	-

**Table 4:** Material properties of ceramics.

Parameter	Value	Unit
Young’s modulus *E*	3.7 $\times 10^{12}$	Pa
Poisson’s ratio ν	0.33	–
Density ρ	3980	kg/m^3^
Thermal conductivity *k*	31	J/(s· m· K)
Specific heat capacity *c*	880	J/(kg · K)
Thermal expansion coefficient α	7.5 $\times 10^{-6}$	1/K
Fracture energy *G*	42.47	N/m
Convective heat transfer coefficient $h_{s}$	65,000 ($T_{0}=300$ °C)	W/m^2^ · K
	90,000 ($T_{0}=350$ °C)	
	82,000 ($T_{0}=400$ °C)	
	70,000 ($T_{0}=500$ °C)	
	60,000 ($T_{0}=600$ °C)	

## Data Availability

The original contributions presented in this study are included in the article. Further inquiries can be directed to the corresponding author.

## Abbreviations

The following abbreviations are used in this manuscript:

PD	Peridynamics
CCM	Classical continuum mechanics
XFEM	Extended finite element method
PFM	Phase-field fracture method
DEM	Discrete element method
BB-PD	Bond-based peridynamics
OSB-PD	Ordinary state-based peridynamics
NOSB-PD	Non-ordinary state-based peridynamics

## References

[1] Belytschko T., Black T. (1999). Elastic Crack Growth in Finite Elements with Minimal Remeshing. Int. J. Numer. Methods Eng..

[2] Francfort G.A., Marigo J.J. (1998). Revisiting Brittle Fracture as an Energy Minimization Problem. J. Mech. Phys. Solids.

[3] Cundall P.A. A Computer Model for Simulating Progressive Large-Scale Movements in Blocky Rock Systems. Proceedings of the ISRM International Symposium.

[4] Jaskowiec J., Plucinski P., Pamin J. (2015). Thermo-Mechanical XFEM-type Modeling of Laminated Structure with Thin Inner Layer. Eng. Struct..

[5] Kumar R., Lal A., Sutaria B.M., Magar A. (2024). Thermo-mechanical fracture analysis of porous functionally graded cracked plate using XFEM. Mech. Based Des. Struct. Mach..

[6] Badnava H., Msekh M.A., Etemadi E., Rabczuk T. (2018). An H-Adaptive Thermo-Mechanical Phase Field Model for Fracture. Finite Elem. Anal. Des..

[7] Zhou H., Tian X., Wu J. (2024). Cracking and Thermal Resistance in Concrete: Coupled Thermo-Mechanics and Phase-Field Modeling. Theor. Appl. Fract. Mech..

[8] Sun L., Liu Q., Tao S., Grasselli G. (2022). A Novel Low-Temperature Thermo-Mechanical Coupling Model for Frost Cracking Simulation Using the Finite-Discrete Element Method. Comput. Geotech..

[9] Silling S.A. (2000). Reformulation of Elasticity Theory for Discontinuities and Long-Range Forces. J. Mech. Phys. Solids.

[10] Silling S.A., Askari E. (2005). A meshfree method based on the peridynamic model of solid mechanics. Comput. Struct..

[11] Silling S.A., Epton M., Weckner O., Xu J., Askari E. (2007). Peridynamic States and Constitutive Modeling. J. Elast..

[12] Silling S.A. (2010). Linearized Theory of Peridynamic States. J. Elast..

[13] Bobaru F., Duangpanya M. (2012). A Peridynamic Formulation for Transient Heat Conduction in Bodies with Evolving Discontinuities. J. Comput. Phys..

[14] Oterkus S., Madenci E., Agwai A. (2014). Peridynamic Thermal Diffusion. J. Comput. Phys..

[15] Oterkus S., Madenci E., Agwai A. (2014). Fully Coupled Peridynamic Thermomechanics. J. Mech. Phys. Solids.

[16] Wang S., Zhang X., Li K., Tang J., Feng H., Cheng Z. (2024). Thermo-mechanical coupled peridynamics simulation of concrete failure under fire scenarios. Eng. Fract. Mech..

[17] Zhang G., Dai Z. (2024). A Fully Coupled Thermomechanical Peridynamic Model for Rock Fracturing under Blast Loading Considering Initial Pore Damage. Comput. Geotech..

[18] Cheng Z., Ren X., Zhang J., Zhang X. (2024). Peridynamics Thermomechanical Coupling Simulation of Damage in Engineered Cementitious Composite-Concrete Bonding Specimens under High Temperature. Eng. Fract. Mech..

[19] Sun W., Lu W., Bao F., Ni P. (2021). A PD-FEM Coupling Approach for Modeling Thermal Fractures in Brittle Solids. Theor. Appl. Fract. Mech..

[20] Silling S.A., Lehoucq R.B., Aref H., van der Giessen E. (2010). Peridynamic Theory of Solid Mechanics. Advances in Applied Mechanics.

[21] Lubineau G., Azdoud Y., Han F., Rey C., Askari A. (2012). A morphing strategy to couple non-local to local continuum mechanics. J. Mech. Phys. Solids.

[22] Kilic B., Madenci E. (2010). Peridynamic Theory for Thermomechanical Analysis. IEEE Trans. Adv. Packag..

[23] Liu Z., Liu S., Han F., Chu L. (2022). The Morphing Method to Couple Local and Non-Local Thermomechanics. Comput. Mech..

[24] Timoshenko S.P., Goodier J.N., Abramson H.N. (1970). Theory of Elasticity (3rd Ed.). J. Appl. Mech..

[25] Poirier D.R., Geiger G.H., Poirier D.R., Geiger G.H. (2016). Conduction of Heat in Solids. Transport Phenomena in Materials Processing.

[26] Mandal T.K., Nguyen V.P., Wu J.Y., Nguyen-Thanh C., de Vaucorbeil A. (2021). Fracture of thermo-elastic solids: Phase-field modeling and new results with an efficient monolithic solver. Comput. Methods Appl. Mech. Eng..

[27] Duflot M. (2008). The extended finite element method in thermoelastic fracture mechanics. Int. J. Numer. Methods Eng..

[28] Pham M.V., Nguyen M.N., Bui T.Q. (2025). An adaptive mesh refinement algorithm for crack propagation with an enhanced thermal–mechanical local damage model. Finite Elem. Anal. Des..

[29] Prasad N.N.V., Aliabadi M.H., Rooke D.P. (1994). Incremental crack growth in thermoelastic problems. Int. J. Fract..

[30] Sarkar S., Singh I.V., Mishra B.K. (2020). A Thermo-mechanical Gradient Enhanced Damage Method for Fracture. Comput. Mech..

[31] Jiang C.P., Wu X.F., Li J., Song F., Shao Y.F., Xu X.H., Yan P. (2012). A study of the mechanism of formation and numerical simulations of crack patterns in ceramics subjected to thermal shock. Acta Mater..

[32] Li J., Song F., Jiang C. (2015). A non-local approach to crack process modeling in ceramic materials subjected to thermal shock. Eng. Fract. Mech..

